# Assessment of aPTT-based clot waveform analysis for the detection of haemostatic changes in different types of infections

**DOI:** 10.1038/s41598-020-71063-1

**Published:** 2020-08-25

**Authors:** Chuen Wen Tan, Wan Hui Wong, McVin Hua Heng Cheen, Yvonne Miao Hui Chu, Shan Shan Lim, Lawrence Cheng Kiat Ng, Dillon Guo Dong Yeo, Gayathry Morvil, Lai Heng Lee, Heng Joo Ng

**Affiliations:** 1 Department of Haematology, Singapore General Hospital, Outram Road, Singapore, 169608 Singapore; 2 Department of Pharmacy, Singapore General Hospital, Singapore, Singapore

**Keywords:** Infectious diseases, Biomarkers

## Abstract

Infections cause varying degrees of haemostatic dysfunction which can be detected by clot waveform analysis (CWA), a global haemostatic marker. CWA has been shown to predict poor outcomes in severe infections with disseminated intravascular coagulopathy. The effect of less severe bacterial and viral infections on CWA has not been established. We hypothesized that different infections influence CWA distinctively. Patients admitted with bacterial infections, dengue and upper respiratory tract viral infections were recruited if they had an activated partial thromboplastin time (aPTT) measured on admission. APTT-based CWA was performed on Sysmex CS2100i automated analyser using Dade Actin FSL reagent. CWA parameters [(maximum velocity (min1), maximum acceleration (min2) and maximum deceleration (max2)] were compared against control patients. Infected patients (n = 101) had longer aPTT than controls (n = 112) (34.37 ± 7.72 s vs 27.80 ± 1.59 s, p < 0.001), with the mean (± SD) aPTT longest in dengue infection (n = 36) (37.99 ± 7.93 s), followed by bacterial infection (n = 52) (33.96 ± 7.33 s) and respiratory viral infection (n = 13) (29.98 ± 3.92 s). Compared to controls (min1; min2; max2) (5.53 ± 1.16%/s; 0.89 ± 0.19%/s^2^; 0.74 ± 0.16%/s^2^), bacterial infection has higher CWA results (6.92 ± 1.60%/s; 1.04 ± 0.28%/s^2^; 0.82 ± 0.24%/s^2^, all p < 0.05); dengue infection has significantly lower CWA values (3.93 ± 1.32%/s; 0.57 ± 0.17%/s^2^; 0.43 ± 0.14%/s^2^, all p < 0.001) whilst respiratory virus infection has similar results (6.19 ± 1.32%/s; 0.95 ± 0.21%/s^2^; 0.73 ± 0.18%/s^2^, all p > 0.05). CWA parameters demonstrated positive correlation with C-reactive protein levels (min1: r = 0.54, min2: r = 0.44, max2: r = 0.34; all p < 0.01). Different infections affect CWA distinctively. CWA could provide information on the haemostatic milieu triggered by infection and further studies are needed to better define its application in this area.

## Introduction

Infections, with or without sepsis can cause varying degrees of haemostatic dysfunction ranging from subclinical coagulopathy to disseminated intravascular coagulation (DIC) through several mechanisms. Bacterial infections often result in a prothrombotic state due to the release of toxins and cytokines. The ensuing activation of coagulation pathways is supplemented by the aberrant expression of tissue factor on monocytes and macrophages with impairment of anticoagulant systems orchestrated by damaged endothelial cells and the suppression of fibrinolysis^1–5^. In contrast, infections can also give rise to a primary bleeding tendency, with dengue infection being a classic example. The underlying pathophysiology of this bleeding tendency is again likely to be multifactorial. In addition to thrombocytopenia, there are various changes in plasma haemostatic factors with variable reduction in both clotting and anticoagulant factors resulting in decreased thrombin generation and increased fibrinolysis^6–8^.

Clot waveform analysis (CWA) is an extended interrogation of the curve generated by an optical detection system during the measurement of two routine coagulation assays, the prothrombin time (PT) and the activated partial thromboplastin time (aPTT). The system detects either light transmittance or absorbance. CWA is touted to be a global haemostatic assay, reflecting the overall effects of all the haemostatic factors^9^. It therefore provides additional information on the coagulation processes while measuring the PT and aPTT and can be conveniently obtained using special software in coagulation analyzers. Both qualitative (waveform pattern) and quantitative (various calculations derived from the curve) parameters can be generated and analyzed.

The biphasic waveform detected in CWA has been studied extensively in the setting of DIC arising from different causes including infection. This biphasic waveform is caused by the formation of calcium-dependent precipitates of very low-density lipoprotein and C-reactive protein (CRP) upon recalcification of plasma in vitro^10^. This specific waveform if present, has been demonstrated to be an early marker of DIC and was correlated with poor clinical outcomes especially in intensive care settings^10–16^. The effect of less severe infections without DIC on CWA is less known. Considering the myriad of changes affecting haemostasis associated with most infections, effects on CWA parameters are highly conceivable even in less severe or early stages of infections. Peculiar effects on haemostasis by different infective agents may also give rise to specific CWA patterns that permit their identification with the rationalization of treatment for improved outcomes. Therefore, we hypothesized that bacterial and viral infections such as dengue and other common respiratory tract viruses, would generate different CWA parameters from each other and from patients without active infection. This paper reports the findings of our study to investigate this hypothesis. In addition, the relationship between CWA and existing biological markers in infections (CRP and procalcitonin) was explored.

## Materials and methods

### Study design

This study was carried out in a 1,600-bed academic medical centre with a comprehensive clinical and laboratory haematology service between October 2015 and August 2017. The study protocol was approved by the Singhealth Centralised Institutional Review Board with waiver of consent granted and conducted in compliance with the ethical standards of the responsible committee on human experimentation (institutional and national) and with the Helsinki Declaration of 1975, as revised in 2008 (5). aPTT-based CWA parameters of study subjects with and without infections were retrieved from our laboratory data storage system and analyzed for comparison. The identification and selection of study subjects were as follows:

#### Controls

Patients admitted for elective orthopaedic and urological procedures between October 2015 and January 2016 were identified daily through admission records and screened if they had an aPTT performed as part of their pre-operative investigations. Only data from their pre-operative aPTT were used in this study. Patients were excluded if they had active cancer, were taking an anticoagulant or had a venous or arterial thrombotic event within the last 90 days before admission.

#### Patients with infection

Admission records of our General Medicine Department were screened during each study period to identify patients admitted with a suspected infection. Further screening was performed if they had aPTT specimen sent by the clinical team within 48 h of the admission. If more than one aPTT test was sent, CWA from the first aPTT was used. The same exclusion criteria for controls were applied to these study subjects. Final selection of subjects was made upon a definitive diagnosis of an infection. These patients were then grouped according to the type of infections, namely bacterial, dengue and viral respiratory tract infection.

Patients with proven bacterial infection were further subdivided into those with and without bacteremia. The former was defined as patients having a positive blood culture performed within 48 h of the admission and the latter was defined as those with a positive bacterial culture from urine, sputum or stool culture specimen collected within 48 h of the admission but with negative blood culture specimens.

For the group with dengue infection, the diagnosis must be confirmed by a positive result of any of the following tests performed within 48 h of admission: dengue IgM antibody, dengue NS1 antigen and dengue PCR. They must not have any other positive viral or bacterial tests.

The viral respiratory tract infection group was defined as patients with a positive respiratory tract virus test result performed within 48 h of admission. They must not have any evidence of bacterial infection from either blood and sputum culture.

Baseline demographic and clinical data of all subjects and controls were collected upon their selection and recruitment. In addition, CRP and/or procalcitonin results amongst the infected patients performed within 24 h from the time of performance of the aPTT were recorded.

### Outcome measures and collection of CWA data

aPTT-based CWA data were retrieved from the CS2100i automated coagulation analyser (Sysmex Corporation, Kobe, Japan). The Dade Actin FSL reagent (Siemens Healthcare, Marburg, Germany) used for the determination of aPTT contains purified soy phosphatides and rabbit brain phosphatides in 1.0 × 10^–4^ M ellagic acid. A built-in algorithm tool on the analyzer generates the clotting curve measured at 660 nm and three other CWA parameters – maximum velocity (min1), maximum acceleration (min2), maximum deceleration (max2) and the corresponding timings (Tmin1, Tmin2, Tmax2)^17^. The parameters used for comparison between the groups were the subjects’ aPTT, min1, min2 and max2 values.

### Statistical analysis

Descriptive parameters were presented as mean [standard deviation (SD)] and n (%) for numerical and categorical variables respectively. We compared characteristics of patients and controls using two-sample *t* test or Mann–Whitney *U* test and chi-square or Fisher’s exact test for continuous and categorical variables respectively. The CWA parameters were compared between groups using one-way analysis of variance, followed by multiple linear regression to adjust for differences in age, gender and ethnicity. Additionally, we used multiple linear regression to identify factors significantly associated with changes in CWA parameters. We used Pearson’s correlation analysis to evaluate the relationship between CWA parameters and CRP and procalcitonin, respectively. All analyses were performed using IBM SPSS Statistics for Windows, version 24 (IBM Corporation, Armonk, New York, USA) at the 5% significance level.

## Results

A total of 101 patients with infections and 112 controls were recruited. There were 52 patients with bacterial infection (51.5%), 36 with dengue (35.6%) and 13 with respiratory viral infection (12.9%). The characteristics of the subjects are summarized in Table 1.

**Table 1:** Characteristics of patients with infections and controls.

Characteristics	Bacterial infection (n = 52)	Dengue infection (n = 36)	Other viral infection (n = 13)	Control (n = 112)	p-value		
					Bacterial vs. control	Dengue vs. control	Other virus vs. control
Age, mean ± SD (years)	64.8 ± 14.4	51.0 ± 14.5	82.1 ± 9.0	65.1 ± 11.0	NS	< 0.001	< 0.001
Male, n (%)	33 (63.5)	24 (66.7)	6 (46.2)	41 (36.6)	0.001	0.002	NS
**Ethnicity, n (%)**					NS	0.001	NS
Chinese	42 (80.8)	23 (63.9)	9 (69.2)	97 (86.6)			
Indian	4 (7.7)	5 (13.9)	0 (0)	6 (5.4)			
Malay	6 (11.5)	2 (5.6)	3 (23.1)	7 (6.2)			
Others	0 (0)	6 (16.7)	1 (7.7)	2 (1.8)			

Post-hoc tests with Bonferroni correction were performed for pairwise comparisons.

*SD* standard deviation, *NS* non-significant (p > 0.05).

Bacterial infections could further be classified into Gram-negative bacteremia (n = 26, 44.1%), Gram-positive bacteremia (n = 17, 28.8%) and non-bacteremic bacterial infection (n = 16, 27.1%). Gram-negative microbes identified were *Aeromonas* species, *Cronobacter sakazakii*, *Enterobacter cloacae complex*, *Enterococcus faecalis*, *Escherichia coli*, *Klebsiella pneumoniae, Moraxella* species, *Proteus mirabilis*, *Providencia stuartii*, *Pseudomonas aeruginosa*, *Salmonella enteritidis*, and *Stenotrophomonas maltophilia*. *Staphylococcus aureus*, *Streptococcus anginosus* and *Group G Streptococcus* were the Gram-positive bacteria causing bacteremia. For the non-bacteremic infections, the bacteria involved were *Escherichia coli*, *Klebsiella pneumoniae, Pseudomonas aeruginosa, Salmonella enteritidis, Serratia marcescens* and *Staphylococcus aureus*. Respiratory viruses identified were *Influenza A, Influenza B, Rhinovirus A/B/C* and *Respiratory Syncytial Virus B*.

The mean aPTT of all 3 infective groups were longer than the controls although it did not reach statistical significance for viral respiratory tract infection (Table 2). Prolongation of the aPTT was however not universal as only 55 patients (54.5% of infected patients) were affected. Amongst those with prolonged aPTT, the mean aPTT was 39.0 s with a range of 33.0 s to 72.4 s (normal range 25.7 s to 32.9 s).

**Table 2:** Comparison of clot waveform parameters between infected and control blood samples.

CWA Parameter^a^	Bacterial infection (n = 52)	Dengue infection (n = 36)	Other viral infection (n = 13)	Control (n = 112)	p-value (p-value after adjustment*)					
					Bacteria vs. control	Dengue vs. control	Other virus vs. control	Bacteria vs. dengue	Dengue vs. viral	Other virus vs. bacteria
aPTT (s)	32.96 ± 7.33	37.99 ± 7.93	29.99 ± 3.92	27.80 ± 1.59	< 0.001 (< 0.001)	< 0.001 (< 0.001)	NS (NS)	0.005 (0.002)	0.003 (0.007)	NS (NS)
Min1 (%/s)	6.92 ± 1.60	3.93 ± 1.02	6.19 ± 1.32	5.53 ± 1.16	< 0.001 (< 0.001)	< 0.001 (< 0.001)	NS (NS)	< 0.001 (< 0.001)	< 0.001 (< 0.001)	NS (NS)
Min2 (%/s^2^)	1.04 ± 0.28	0.57 ± 0.17	0.95 ± 0.21	0.93 ± 0.46	< 0.001 (< 0.001)	< 0.001 (< 0.001)	NS (NS)	< 0.001 (< 0.001)	< 0.001 (0.001)	NS (NS)
Max2 (%/s^2^)	0.82 ± 0.24	0.43 ± 0.14	0.73 ± 0.18	0.74 ± 0.16	0.036 (0.030)	< 0.001 (< 0.001)	NS (NS)	< 0.001 (< 0.001)	< 0.001 (0.003)	NS (NS)

Post-hoc tests with Bonferroni correction were performed for pairwise comparisons.

*CWA* clot waveform analysis, *SD* standard deviation, *NS* non-significant (p > 0.05).

*Adjusted for age, gender and ethnicity.

^a^All CWA results are expressed in mean ± SD.

**Figure 1:**
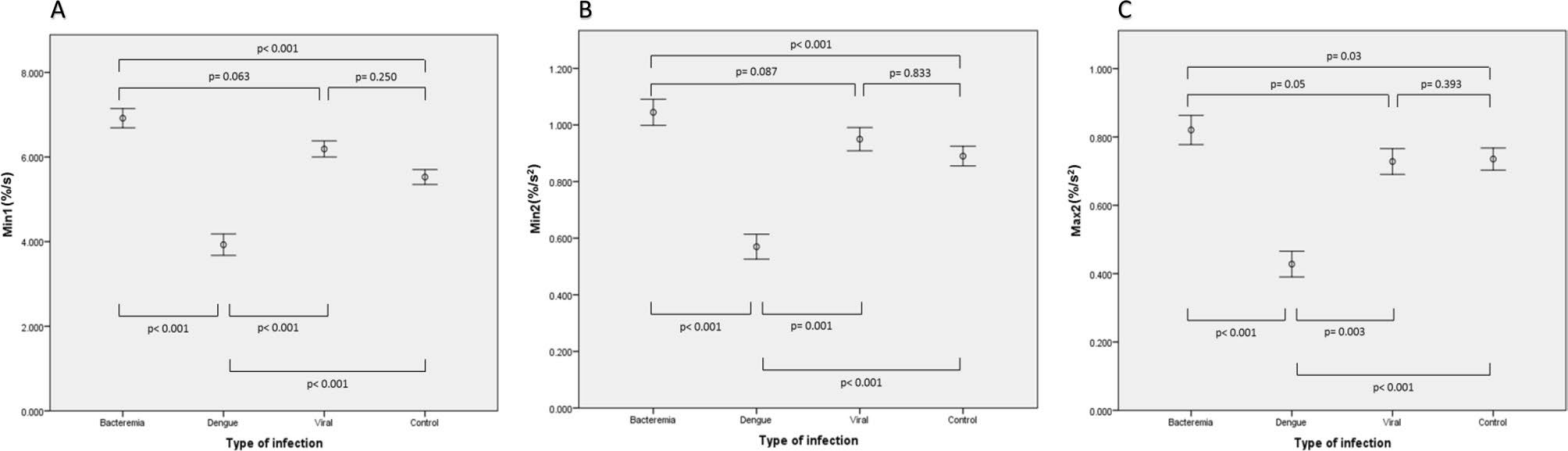
Comparison of clot waveform analysis (CWA) parameters between types of infection and control. Comparison of CWA parameters amongst study subjects with (**A**)–(**C**) demonstrating the min1, min2 and max2 results respectively. All p-values are adjusted for age, gender and ethnicity.

Comparison of CWA parameters between groups are shown in Table 2 and Fig. 1. No patient exhibited a biphasic waveform. Patients with bacterial infections generated significantly higher CWA parameters compared with controls. In contrast, patients with dengue infection yielded significantly lower CWA parameters. Those with other viral respiratory infections however showed no difference in CWA parameters from controls. Therefore, all the CWA parameters (both adjusted and unadjusted) in patients with dengue infections were significantly lower than those with bacterial infection (p < 0.05) and other viral infection (p < 0.001). Although the bacterial infection group had higher CWA parameters than the group with other viral respiratory infection, the differences between these groups were not statistically significant (p-value of all the parameters > 0.05) (results available in Supplementary Materials).

To ensure that sensitivity of the analysis was retained, the 46 patients with normal baseline aPTT results were analyzed separately and demonstrated CWA findings similar as reported above (results available in Supplementary Materials). Sub-group analysis (divided into Gram positive bacteremia, Gram negative bacteremia and non-bacteremic groups) of those with bacterial infections showed no significant differences in CWA parameters across the three groups except for max2 between Gram-positive and the non-bacteremic groups (results available under Supplementary Materials).

Approximately two thirds of the infected patients had their CRP and/or procalcitonin data available (Table 3). Min1, min2 and max2, but not aPTT, of patients with infection demonstrated weak to moderate positive correlation with CRP levels (min1: r = 0.540, p < 0.001; min2: r = 0.439, p < 0.001; max2: r = 0.340, p = 0.004; aPTT: r = 0.118, p = 0.331). On the other hand, neither CWA parameters nor aPTT correlated with procalcitonin (min1: r = 0.180, p = 0.137; min2: r = 0.129, p = 0.288; max2: r = 0.102, p = 0.402; aPTT: r = 0.119, p = 0.325).

**Table 3:** C-reactive protein and procalcitonin data amongst the patients with infection.

Type of infection	CRP (mg/L)		Procalcitonin (ng/mL)	
	No. of cases with data	Mean ± SD	No. of cases with data	Mean ± SD
Bacterial infection	42	155.3 ± 95.6	40	28.1 ± 41.3
Dengue infection	14	16.2 ± 14.4	12	0.4 ± 0.2
Other viral infections	8	55.9 ± 39.9	11	0.8 ± 1.3

*CRP* C-reactive protein, *SD* standard deviation.

## Discussion

Our study has reaffirmed the notion that infections can cause significant changes to haemostatic mechanisms even in the absence of DIC and these alterations could be detected by CWA. Previous studies on CWA have mainly focused on the biphasic waveform which is observed in patients with sepsis and is a marker of early DIC^18,19^. In this study we have shown that aPTT-based CWA as a global haemostatic marker, is sufficiently sensitive to detect changes in haemostatic function from less severe infections by bacterial organisms and in dengue. These changes in CWA parameters were present irrespective of the underlying aPTT result.

Significantly, the effects of bacterial and dengue infections on aPTT-based CWA parameters were distinctive and divergent. This is in contrast to the routine aPTT results in which all the infected groups exhibited a non-discriminatory longer mean clotting time than controls. Patients with bacterial infections exhibited significantly higher min1, min2 and max2 compared with controls after adjusting for age, gender and ethnicity. These first and second derivatives of the original transmittance curve have been previously hypothesized to represent thrombin and prothrombinase activities respectively^20^. Raised CWA parameters have also recently been demonstrated to be associated with venous thromboembolism^17^. It can therefore be deduced that bacterial infections lead to an overall prothrombotic state or activation of coagulation system from the CWA parameter changes. This is consistent with the findings of other studies^1–4^ which had shown bacterial infections resulting in activation of the coagulation system and impairment of natural anticoagulant and fibrinolytic pathways.

In contrast, patients having dengue infections demonstrated significantly lower CWA parameters than controls. This pattern of alteration in CWA implies a reduction in the global haemostatic functions and is in keeping with the general clinical features observed in dengue infection where bleeding is a potential complication. Our findings are therefore consistent with existing data indicating thrombin generation is reduced in dengue^6^ and dengue virus nonstructural protein NS1 can inhibit prothrombin activation^21^. In addition, bleeding in dengue infection may also result from increased fibrinolysis^6^. Clot-fibrinolysis waveform analysis (CFWA) is a novel extension of CWA-based method using both aPTT reagent and tissue plasminogen activator for simultaneous assessment of both clot formation and fibrinolysis^22^ and application of CFWA in future studies might be very useful in assessing the bleeding risk in dengue more holistically.

Although there are evidence linking viral respiratory infection with arterial thrombotic events^23^, our data demonstrated that common respiratory viral infections did not significantly affect CWA parameters. It has previously been described that acute respiratory tract infections could cause changes in haemostatic markers, including thrombin generation assay, with the overall effect depicting a procoagulant state^24,25^. Although we observed a trend towards increased CWA parameters especially min1, amongst our patients with viral respiratory tract infection, these differences did not reach statistical significance. It is plausible that CWA might not be sensitive in detecting these subtle changes in coagulation or our sample size might not have the statistical power to demonstrate these differences.

As an acute phase reactant, CRP is not a specific infection marker in isolation and can be elevated in various inflammatory and infective conditions^26^. Our study demonstrated that there is statistically significant weak to moderate correlation between CWA parameters and CRP levels. Interleukin 6 (IL-6) stimulates the production of CRP in the liver and IL-6 together with other pro-inflammatory cytokines could upregulate the synthesis of some coagulation factors^27–29^. We postulate that some of the changes in CWA observed could be partially accounted for by changes in other acute phase reactants that could potentially influence coagulation such as factor VIII, prothrombin and fibrinogen, although these data were not available in the present study. The absence of a correlation between aPTT and CRP suggests that CWA is more sensitive than aPTT in detecting haemostatic changes in this clinical setting. As CWA measures only haemostasis, its relationship with CRP may be diluted by the additional effects of inflammation^30–34^ resulting in the at best moderate correlation with CRP. On the other hand, CWA parameters did not correlate with procalcitonin. Although the definitive explanations for this observation remain uncertain and could be an area for future exploration, possible postulations include procalcitonin, unlike CRP, is a marker more specific for bacterial infection than for proinflammatory conditions in general and procalcitonin changes more rapidly than CRP does^35^ and thus CWA parameters might not parallel the fluctuations in procalcitonin well.

As CWA is derived from the process of fibrin clot formation, its parameters are influenced by fibrinogen concentrations. Even though the fibrinogen levels were not measured in study, if fibrinogen is indeed the main driver for the increased CWA parameters shown in the cases with bacterial infection, it would be expected that these cases would have either normal or slightly shortened aPTT. On the contrary, our cases with bacterial infection had longer clotting time compared to the controls. In fact, when compared to controls, all the infected cases demonstrated a non-discriminatory longer clotting time but their CWA parameters moved in different directions. Unfortunately, the design of the present study does not permit us to address the underlying mechanism for these distinctive CWA patterns observed in different infections. Correlation with other coagulation markers such as factor VIII, fibrinogen and D-dimer, and inflammatory markers such as C-reactive protein and procalcitonin, in a larger and prospective patient cohort would be very interesting and useful to provide some answers to our present observations.

Our comparison of the CWA parameters between the three infective groups could give rise to various potential clinical uses. Firstly, in patients presenting with febrile illnesses, CWA parameters could provide an additional marker to help distinguish bacterial infections from viral respiratory illnesses. This may guide the use and early administration of antibiotics to those with prolonged CWA parameters. CWA could therefore complement or replace existing pro-inflammatory markers such as C-reactive protein and procalcitonin in the diagnosis of bacterial infection and sepsis. On the other hand, in patients with low CWA parameters, a consideration has to be made for the possibility of dengue fever in the appropriate clinical setting. Since CWA results may be available prior to the availability of confirmatory test results for dengue, this may allow for stratification of patients for appropriate monitoring or escalation of care. Secondly, this study supports additional efforts to determine if CWA changes could be correlated with disease severity and prognosis. Previous studies looking at biphasic waveform, a derivative of CWA, in sepsis had established that it preceded the clinical occurrence of DIC and served as a prognostic marker for ICU stay, longer hospitalization and increased mortality^11,15,16,36,37^. Hence, CWA parameters could be studied to determine if they have similar value in less severe infections. As CWA parameters denote either a hypercoagulable or hypocoagulable state, correlating them with incidence of thrombosis or bleeding in infected patients could help determine the role of thromboprophylaxis or prophylactic measures against bleeding.

An attractive feature of aPTT-based CWA parameters is that it is an extension of the routine aPTT test and comes with no extra cost or testing time whenever an aPTT is performed. It is therefore readily available and is inexpensive compared to other inflammatory markers such as C-reactive protein and procalcitonin. CWA can indeed be a valuable tool in resource poor settings to assist clinicians in differentiating various infections and thus initiating appropriate timely management.

One drawback of using CWA may be its perceived complexity because of the number of parameters measured. This however can be addressed by utilizing fewer parameters, making its interpretation less daunting. Based on our findings, min1 appeared to be the most discriminatory for the various infection groups studied. Min1 was consistently highest amongst patients with bacterial infection followed by viral respiratory tract infection, controls and dengue infection. Most of the differences in min1 observed across the groups were highly statistically significant and remained so irrespective of their underlying aPTT status. As such, a single CWA parameter can be made available to clinicians which will simplify its understanding and help promote its adoption.

Our current study however has a number of limitations. Although the types of bacterial organism identified in this study is extensive, it is not exhaustive. Hence, the effects of bacterial infections on CWA shown here may not necessarily be extrapolated to all types of bacterial infections. Distinction between Gram-negative organisms which are more likely to give rise to severe sepsis from the Gram-positives also cannot be made based on the small numbers recruited into each category. For the hypocoagulable state, we could only demonstrate this phenomenon in dengue infection which is endemic in our region. The effects of other haemorrhagic viral infections found in other parts of the world on CWA parameters cannot be studied in our present location. Similarly, we cannot conclusively discount the value of CWA in many other viral infections despite not demonstrating an effect among infections due to respiratory viruses. Also, the dynamic CWA changes over the course of infection within an individual patient and the influence of different infections on CWA triggered by different coagulation reagents was not evaluated in our present study. These limitations can only be further addressed by more extensive and similarly well-controlled studies which we hope to provide impetus with our present effort.

In conclusion, we demonstrated that bacterial, dengue and viral respiratory tract infections give rise to distinctive aPTT-based CWA parameters. CWA could provide additional information on the haemostatic milieu triggered by infection than the routine coagulation assays. Further efforts will be needed to validate these findings and refine the clinical application of CWA in this area.

## Supplementary information

Supplementary Tables.
