# Supplementary material for: Assessment of aPTT-based clot waveform analysis for the detection of haemostatic changes in different types of infections
Source: Sci Rep. 2020 Aug 25;10:14186. doi: 10.1038/s41598-020-71063-1 (PMC7447776; doi:10.1038/s41598-020-71063-1)
Supplement: Supplementary file 1 — Supplementary Tables. [file 41598_2020_71063_MOESM1_ESM.docx]

**Assessment of aPTT-based clot waveform analysis for the detection of haemostatic changes in different types of infections**

**Authors and affiliations:**

Chuen Wen Tan*^1^, Wan Hui Wong^1^, McVin Hua Heng Cheen^2^, Yvonne Miao Hui Chu^1^, Shan Shan Lim^1^, Lawrence Cheng Kiat Ng^1^, Dillon Guo Dong Yeo^1^, Gayathry Morvil^1^, Lai Heng Lee^1^, Heng Joo Ng^1^

^1^Department of Haematology, Singapore General Hospital, Singapore

^2^Department of Pharmacy, Singapore General Hospital, Singapore

***Corresponding author:**

Chuen Wen Tan

Department of Haematology,

Singapore General Hospital,

Outram Road,

Singapore 169608.

Email: [tan.chuen.wen@singhealth.com.sg](mailto:tan.chuen.wen@singhealth.com.sg)

**Running head:** Clot waveform analysis and infections

**Conflict of Interest:** None for all the authors.

Supplementary Table 1: Comparison of clot waveform parameters between patients with infection and controls with normal^ aPTT results.

| **CWA Parameter^#^** | **Patient Group** | | | | **p-value (p-value after adjustment*)** | | |
| --- | --- | --- | --- | --- | --- | --- | --- |
|  | **Bacterial infection**  **(n= 30)** | **Dengue infection**  **(n= 6)** | **Other viral infection**  **(n= 10)** | **Control**  **(n= 112)** | **Bacterial vs. Control** | **Dengue vs. Control** | **Other virus vs. Control** |
| **Min1 (%/s)** | 7.20 ± 1.54 | 4.40 ± 0.43 | 6.12 ± 1.50 | 5.53 ± 1.16 | <0.001 (<0.001) | NS (0.050) | NS (NS) |
| **Min2 (%/s^2^)** | 1.13 ± 0.25 | 0.76 ± 0.06 | 0.95 ± 0.24 | 0.89 ± 0.19 | <0.001 (<0.001) | NS (0.054) | NS (NS) |
| **Max2 (%/s^2^)** | 0.90 ± 0.22 | 0.57 ± 0.04 | 0.75 ± 0.19 | 0.74 ± 0.16 | <0.001 (<0.001) | NS (0.035) | NS (NS) |

Normal^ – included aPTT results both within and shorter than normal range.

^#^All CWA results are expressed in mean ± SD.

*Adjusted for age, gender and ethnicity.

Post-hoc tests with Bonferroni correction were performed for pairwise comparisons.

CWA – clot waveform analysis; SD – standard deviation; NS – Non-significant (p > 0.05).

Supplementary Table 2: Comparison of clot waveform parameters amongst the different subgroups of bacterial infection.

|  |  |  |  | **p-value (p-value after adjustment*)** | | |
| --- | --- | --- | --- | --- | --- | --- |
| **CWA**  **Parameter^** | **Gram negative (n= 23)** | **Gram positive**  **(n= 14)** | **Not bacteremic (n= 15)** | **Gram positive vs.**  **Gram negative** | **Gram positive vs.**  **Not bacteremic** | **Gram negative vs.**  **Not bacteremic** |
| **Min1 (%/s)** | 7.17 ± 1.39 | 6.27 ± 1.57 | 7.14 ± 1.86 | NS (NS) | NS (NS) | NS (NS) |
| **Min2 (%/s^2^)** | 1.06 ± 0.26 | 0.93 ± 0.25 | 1.13 ± 0.31 | NS (NS) | NS (NS) | NS (NS) |
| **Max2 (%/s^2^)** | 0.82 ± 0.23 | 0.72 ± 0.20 | 0.91 ± 0.28 | NS (NS) | NS (0.034) | NS (NS) |

^All CWA results are expressed in mean ± SD.

*Adjusted for age, gender and ethnicity.

Post-hoc tests with Bonferroni correction were performed for pairwise comparisons.

CWA – clot waveform analysis; SD – standard deviation; NS – Non-significant (p > 0.05).
